# Supplementary material for: Resolving the full spectrum of human genome variation using Linked-Reads
Source: Genome Res. 2019 Apr;29(4):635–45. doi: 10.1101/gr.234443.118 (PMC6442396; doi:10.1101/gr.234443.118)
Supplement: Supplemental Material [file supp_gr.234443.118_Supplemental_longranger-2.2.zip › longranger-2.2.tar.bz2!/lr2.2-public/lib/stan/cmdstan/stan/doc/api/html/agrad_2rev_2functions_2inv_8hpp_source.html]

Stan: src/stan/agrad/rev/functions/inv.hpp Source File


|  |  |
| --- | --- |
|  | Stan  2.5.0  probability, sampling & optimization |


- Main Page
- Related Pages
- Namespaces
- Classes
- Files

- File List
- File Members

All Classes Namespaces Files Functions Variables Typedefs Enumerations Enumerator Friends Macros Pages

- src
- stan
- agrad
- rev
- functions

inv.hpp

Go to the documentation of this file.

1 #ifndef STAN\_\_AGRAD\_\_REV\_\_FUNCTIONS\_\_INV\_HPP

2 #define STAN\_\_AGRAD\_\_REV\_\_FUNCTIONS\_\_INV\_HPP

3

4 #include <valarray>

5 #include <stan/agrad/rev/var.hpp>

6 #include <stan/agrad/rev/internal/v\_vari.hpp>

7 #include <stan/math/functions/inv.hpp>

8

9 namespace stan {

10  namespace agrad {

11

12  namespace {

13  class inv\_vari : public op\_v\_vari {

14  public:

15  inv\_vari(vari\* avi) :

16  op\_v\_vari(stan::math::inv(avi->val\_),avi) {

17  }

18  void chain() {

19  avi\_->adj\_ -= adj\_ / (avi\_->val\_ \* avi\_->val\_);

20  }

21  };

22  }

23

43  inline var inv(const var& a) {

44  return var(new inv\_vari(a.vi\_));

45  }

46

47  }

48 }

49 #endif

stan::agrad::var::vi\_

vari \* vi\_

Pointer to the implementation of this variable.

**Definition:** var.hpp:40

stan::agrad::inv

fvar< T > inv(const fvar< T > &x)

**Definition:** inv.hpp:15

inv.hpp

v\_vari.hpp

var.hpp

stan::agrad::var

Independent (input) and dependent (output) variables for gradients.

**Definition:** var.hpp:27


---

[ Stan Home Page ]

*© 2011–2014,
Stan Development Team.*
